# Letter from the Editor-in-Chief

**DOI:** 10.19102/icrm.2017.080902

**Published:** 2017-09-15

**Authors:** Moussa Mansour


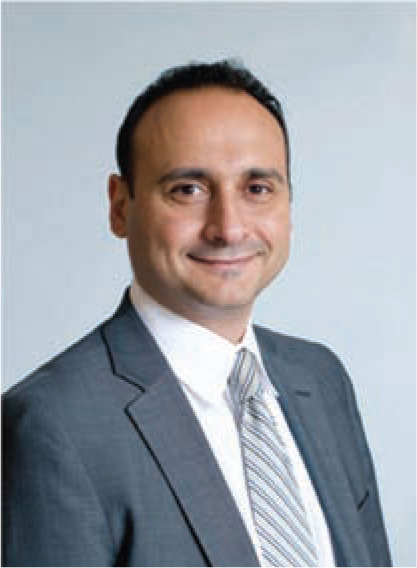


Dear Readers,

We are witnessing the progression of a very interesting era for the specialty of cardiac electrophysiology. The advances that have been made in cardiac monitoring technologies over the past few years have revolutionized our field and have resulted in a significant improvement in our ability to diagnose and treat cardiac arrhythmias, and to prevent some of the major sequalae that can result from them. Wearable and implantable cardiac rhythm recording devices have been miniaturized and/or integrated with mobile phones, allowing for continuous and long-term monitoring of patients. Today’s pacemaker and defibrillators have expanded recording abilities, allowing for the detection of asymptomatic atrial fibrillation and all sorts of other arrhythmias. The availability of new technology, together with an increased awareness of the risk of stroke and atrial fibrillation, has resulted in an explosion in the use of cardiac monitoring devices. This phenomenon is certainly expected to increase our ability to detect atrial fibrillation and reduce the rate of stroke, as demonstrated in some early studies, for example CRYSTAL-AF, that show a positive correlation between the duration of AF monitoring and cryptogenic stroke.

The different types of cardiac monitoring devices and the studies supporting their use have been elegantly summarized in the article by Dr. Chirag Sandesara and colleagues in this issue of *The Journal of Innovations in Cardiac Rhythm Management.* Interestingly, the authors also eluded to another aspect of this monitoring “revolution,” which is the difficulties associated with the handling of the enormous amount of data provided by these devices. All of us are bombarded daily by messages about patients having asymptomatic runs of atrial fibrillation and/or non-sustained ventricular tachycardia. Some device clinics have been overwhelmed by the amount of data being downloaded on a daily basis by the monitoring devices. This problem is exacerbated by the lack of information available to guide the management of these situations: namely, there are no robust studies that specify a certain duration of atrial fibrillation above which the initiation of anticoagulation is recommended. Thus, well-designed clinical studies are urgently needed to generate recommendations to guide the management of patients with monitoring devices. Also needed are changes in the reimbursement system to allow for the hiring of additional human resources—such as data analysists or administrative support—to shoulder some of the work and free up doctors and nurses to perform their job properly.

I hope that you enjoy reading this issue of the *Journal*.

Sincerely,


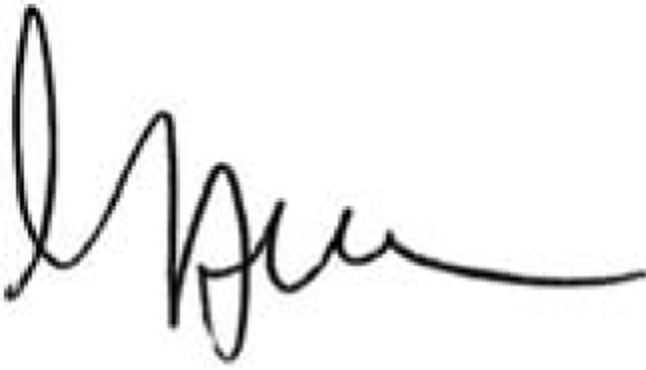


Moussa Mansour, MD, FHRS, FACC

Editor-in-Chief

The Journal of Innovations in Cardiac Rhythm Management

MMansour@InnovationsInCRM.com

Director, Atrial Fibrillation Program

Massachusetts General Hospital

Boston, MA 02114

